# Effect of the Yon PD App on the Management of Self-Care in People With Parkinson Disease: Randomized Controlled Trial

**DOI:** 10.2196/62822

**Published:** 2025-04-02

**Authors:** JuHee Lee, Subin Yoo, Yielin Kim, Eunyoung Kim, Hyeran Park, Young H Sohn, Yun Joong Kim, Seok Jong Chung, Kyoungwon Baik, Kiyeon Kim, Jee-Hye Yoo

**Affiliations:** 1 College of Nursing Mo-Im Kim Nursing Research Institute Yonsei University Seoul Republic of Korea; 2 College of Nursing and Brain Korea 21 FOUR Project Yonsei University Seoul Republic of Korea; 3 Division of Nursing Severance Hospital, Yonsei University Health System Seoul Republic of Korea; 4 Department of Neurology, College of Medicine Yonsei University Seoul Republic of Korea; 5 Department of Neurology, College of Medicine Korea University Seoul Republic of Korea; 6 College of Nursing CHA University Pocheon-si Republic of Korea

**Keywords:** Parkinson disease, mHealth, mobile health, app, neurodegenerative disease, geriatric, quality of life, bradykinesia, nonmotor symptoms, self-care, motor symptoms, cognitive impairment, autonomic, monitoring, management, randomized controlled trial

## Abstract

**Background:**

As the percentage of the older population increases, it is accompanied by an increase in the prevalence of Parkinson disease (PD). People with PD experience a range of nonmotor symptoms, including pain, constipation, dysphagia, sleep disturbances, and fatigue. Improving self-care is necessary for people with PD because it is a chronic disease that requires lifelong management. In our previous study, we developed a mobile app (Yon PD app) to monitor nonmotor symptoms of PD. In this study, we investigated the long-term effects of the app in a larger group of people.

**Objective:**

This study aimed to examine the effectiveness of a mobile app on the management of self-care in people with PD.

**Methods:**

This was a randomized controlled trial. People with PD aged ≥50 years and able to use a smartphone were recruited from the neurology outpatient clinic of a tertiary hospital in South Korea. In total, 102 participants were enrolled in this study. The intervention group was requested to record 5 nonmotor symptoms (pain, constipation, dysphagia, sleep disturbances, and fatigue) for 12 weeks using the mobile app. The control group was requested to record these 5 nonmotor symptoms on a paper questionnaire. General characteristics including age, sex, level of education, disease severity, and comorbidities were examined at baseline. The degree of self-care was examined using the Self-Care of Chronic Illness Inventory at baseline, 6 weeks, and 12 weeks. At 12 weeks, satisfaction with the app was also examined. General characteristics and satisfaction with the app were analyzed using descriptive statistics. The effect of the app on self-care was analyzed using the repeated-measures ANOVA with an α level of .05.

**Results:**

In total, 93 participants were included in the analysis. There were 41 and 52 participants in the intervention and control groups, respectively. The general characteristics of the 2 groups were comparable. Monitoring nonmotor symptoms with the app effectively increased self-care maintenance (*F*_2182_=4.087; *P*=.02) and prevented a decrease in self-care monitoring (*F*_2182_=3.155; *P*=.045). However, using the app was ineffective in improving self-care management (*F*_2182_=1.348; *P*=.26). Self-care management gradually decreased over the 12-week period in both groups. The intervention (n=41) adherence rate reached 60.84% at 6 weeks but decreased to 41.87% by 12 weeks.

**Conclusions:**

Participants were able to improve the degree of self-care by monitoring their nonmotor symptoms using the app. However, additional strategies that increase motivation and enjoyment are required to improve adherence.

**Trial Registration:**

Clinical Research Information Service KCT0006433; https://tinyurl.com/3vmf435m

## Introduction

Parkinson disease (PD) is the second most prevalent neurodegenerative disease, commonly occurring after the age of 65 years [1]. In 2020, the number of people with PD worldwide was estimated to be 3.4 million, which was 1.5 times higher than in 2016 [2]. As the proportion of the older population increases, the proportion of people with PD also increases. PD is an incurable disease caused by irreversible neurological damage, and people with PD are faced with high medical costs and the need to manage their disease symptoms throughout life [3,4]. The total economic burden of PD in the United States is increasing every year and was estimated at US $51.9 billion in 2017 [4]. Previous studies have emphasized the importance of symptom management in PD since job loss, poor quality of life, and lower self-esteem can result from poor daily performance [5,6].

Over several decades, most researchers have focused on the motor symptoms of PD, such as bradykinesia (slow movement), tremor, rigidity, postural instability, and swallowing problems [1]. Motor symptoms have been widely used as diagnostic criteria for PD because they are clearly observed as the disease progresses [3]. However, it has been found that the neurodegenerative changes in PD also manifest as nonmotor symptoms, which prompted increasing interest in these symptoms [7]. The nonmotor symptoms of PD include neurobehavioral changes (eg, depression and cognitive impairment), autonomic failure (eg, urinary dysfunction and abnormal blood pressure), sleep disorders (eg, insomnia and excessive daytime sleeping), sensory impairments (eg, problems with vision and smell), weight loss, and fatigue [3,7]. Nonmotor symptoms can appear throughout the disease trajectory of PD and may even appear before PD is diagnosed [7]. A study found that people with PD experienced, on average, 7 nonmotor symptoms as the disease progressed without realizing that the nonmotor symptoms were related to PD [8].

Managing nonmotor symptoms is as important as managing motor symptoms because nonmotor symptoms can predict the development of motor symptoms [9,10]. Nonmotor symptoms arise from dopaminergic or nondopaminergic pathways, and this pathophysiology influences the risk of developing PD [9]. For example, previous studies have shown that depression increases the risk of PD by 2.4 times [11] and excessive daytime sleepiness increases the risk 3.3-fold [12]. Nonmotor symptoms occur persistently or variably during the progression of PD, decreasing the patient’s quality of life [5,6] and increasing hospitalization rates and medical costs [9]. Therefore, reducing nonmotor symptoms should be considered a major component of the care plan.

Various medications have been used to slow the progression of PD, but it is not feasible to control all nonmotor symptoms with medications [13]. Since nonmotor symptoms can occur consistently throughout the disease course, many researchers have emphasized the importance of self-care for managing these symptoms [9,10,14]. Improving self-care offers several advantages. It enables people with PD to understand the progression of nonmotor symptoms better. It also facilitates the enhancement of daily management strategies, thereby helping to prevent the exacerbation of disease symptoms [14]. According to Riegel and colleagues [15], self-care consists of 3 components: self-care maintenance, self-care monitoring, and self-care management. These 3 components are connected and work together concurrently. Self-care maintenance refers to behaviors that maintain health. Self-care monitoring involves observing for symptom changes. Self-care management is the response to any observed symptom changes [15].

Recent advances in technology have led to the development of numerous mobile apps that assist populations who have chronic diseases with self-care. Mobile-based interventions have significant advantages over paper-based interventions, as they are more portable, allow immediate data storage, and facilitate data transmission and management [16]. These advantages enable health care providers to monitor individuals’ health status and intervene immediately, allowing for long-term disease management [16,17]. Mobile apps have been feasible and effective in promoting self-care in people with heart failure, type 2 diabetes, and cancer [18-20]. For people with PD, mobile apps to help with medication adherence and to assess motor symptoms have been widely used and studied [21,22]. However, studies on self-care in people with PD related to nonmotor symptoms are lacking [23].

To help people with PD with effective self-care, we developed a mobile app (Yon PD app) that can monitor 5 major nonmotor symptoms: pain, constipation, dysphagia, sleep disturbances, and fatigue [24]. A unique aspect of the Yon PD app is that it monitors nonmotor symptoms of PD that are not covered by other mobile apps. In addition, it allows researchers to monitor participants’ records and provide feedback. The app was designed to be user-friendly for easy access and recording. For example, when recording the severity of constipation, example pictures of stool conditions are provided, and people with PD record their conditions by clicking on the corresponding stool picture.

A 6-week pilot study confirmed the feasibility of the Yon PD app [25]. However, the long-term effectiveness of the Yon PD app for larger populations has not been validated. This study examined whether monitoring nonmotor symptoms for 12 weeks using the Yon PD app could improve self-care in people with PD. During the 12-week study period, the intervention group recorded nonmotor symptoms using the app, while the control group recorded nonmotor symptoms using a paper questionnaire. The main difference between the app and the paper questionnaire was that the app enabled researchers to monitor people with PD records regularly and provide feedback. The three hypotheses of this study were as follows: (1) compared to the control group, the intervention group will have higher self-care maintenance scores after 12 weeks; (2) compared to the control group, the intervention group will have higher self-care monitoring scores after 12 weeks; and (3) compared with the control group, the intervention group will have higher self-care management scores after 12 weeks.

## Methods

### Study Design

This was a single-blind randomized controlled trial that adopted a nonequivalent control group, pretest-posttest design [26]. The participants were randomly assigned to either an intervention or control group using computer-generated random numbers. The generated random numbers were matched sequentially to the enrolled participants. If the last digit of the number was even, the participant was assigned to the intervention group; otherwise, the participant was assigned to the control group. The list of random numbers was stored independently on a password-protected computer. Only authorized researchers were able to access to the list. During the study, researchers did not notify the participants whether they were in the intervention or control group. Thus, only the participants were blinded.

This study was designed as a 12-week intervention study to investigate the long-term effects of the Yon PD app. In the literature, a 12-week intervention design has been widely adopted to identify the effectiveness of mobile-based interventions on populations with chronic diseases [27,28]. The findings of previous studies have demonstrated that a 12-week period is sufficient to identify the effectiveness of the intervention. Since a longer intervention period can increase the risk of participant dropout and lower adherence, we adopted a 12-week intervention design for this study. A pretest was given to all groups at the beginning of the study and a posttest was given 6 weeks and 12 weeks after beginning the intervention. Posttest were given twice to prevent participant dropout and assess the intermediate outcomes of the study.

### Study Participants and Sample Size

Study participants were recruited from the neurology outpatient clinic of a large tertiary hospital in Seoul, South Korea. Participants were recruited through advertisements and referrals within the hospital from October 2022 to February 2023. Participants were eligible to participate if they (1) were aged 50 years and older; (2) were clinically diagnosed with PD by a neurology clinician; (3) were able to use a smartphone (eg, text messaging and using the internet); (4) were able to understand the research statement and consent written in Korean; and (5) agreed to participate in this study. Participants were excluded if they (1) had health problems that could significantly affect the nonmotor symptoms of PD; (2) had difficulty manipulating a smartphone due to visual impairment or severe tremor; and (3) had already participated in another intervention study.

The sample size was calculated using the G*Power 3.1 program (Heinrich-Heine-Universität) [29]. The required sample size was 45 for each group with an effect size of 0.6, α level of .05, and power of 0.6. Considering a 20% dropout rate, the total number of participants was calculated as 108 [30].

### Intervention Group

For the intervention group, we provided a mobile app (Yon PD app) to help monitor the nonmotor symptoms of PD. The Yon PD app was a person-centered navigator program that allowed people with PD to monitor their nonmotor symptoms [24]. Using the Yon PD app, people with PD could monitor the presence and severity of pain, constipation, dysphagia, sleep disturbances, and fatigue. Based on user opinions collected from the pilot test [25], we asked the participants to record their pain, constipation, and dysphagia at least twice a week. Sleep disturbances and fatigue were to be recorded at least once a week.

The participants were able to record the severity of symptoms by clicking pictures or dragging visual scales. For example, the severity of constipation was recorded by clicking on the corresponding picture of stool. The severity of pain and fatigue were recorded by dragging scores on visual scales. The Yon PD app was downloadable from the online app store for free. The user interface was simple and intuitive; therefore, the Yon PD app was feasible for people with PD of advanced ages [25].

### Control Group

The control group recorded their nonmotor symptoms on a paper questionnaire, which contained the same items as the Yon PD app. The participants recorded the presence and severity of pain, constipation, dysphagia, sleep disturbance, and fatigue on a printed paper questionnaire by hand. A picture of the visual scale was provided on a paper questionnaire to allow participants to measure the severity of nonmotor symptoms. The control group was asked to record each nonmotor symptom at the same frequency as the intervention group: pain, constipation, and dysphagia at least twice a week and sleep disturbances and fatigue at least once a week. The completed questionnaires were returned at 6-week and 12-week follow-up meetings.

### Study Process

Data were collected by 2 researchers who had been trained before data collection to ensure interreliability. The collected data included general characteristics, nonmotor symptoms of PD, self-care tasks, and satisfaction with the Yon PD app. Data were collected at baseline, 6 weeks after the intervention, and at the end of the intervention (12 weeks).

We collected baseline data on general characteristics using a self-report questionnaire, and disease information was obtained from the electronic medical records. Participants filled out the self-report questionnaire via paper, online, or by mail according to their preference. Six weeks after the intervention, we collected data on the nonmotor symptoms from either the Yon PD apps (intervention group) or the paper questionnaires (control group). Self-care was evaluated at 6 weeks using a paper questionnaire in both the intervention and control groups. Data on nonmotor symptoms and self-care were collected again at 12 weeks in the same manner as at 6 weeks. Satisfaction with the Yon PD app was evaluated only in the intervention group at 12 weeks.

For 12 weeks, 2 researchers checked the use of the Yon PD app to observe the adherence of the intervention group. Data collected by the Yon PD app were automatically transmitted to an online system. By observing the online system, researchers were able to check whether the intervention group used the Yon PD app regularly. If participants in the intervention group did not record anything on the Yon PD app for a week, researchers called them to encourage use of the app and to check if they were having any difficulties using it. If a participant requested help or information on using the app, researchers provided it immediately.

### Measurement Tools

#### General Characteristics

The general characteristics of the participants included sex, age, income, marital status, level of education, presence of a caregiver, living arrangement, employment, disease severity and duration, and the number of comorbidities, which were obtained through a self-report questionnaire. Disease-related information (ie, disease severity, disease duration, and number of comorbidities) was obtained from the electronic medical records.

Disease severity was evaluated by a neurology clinician using the Unified Parkinson Disease’s Rating Scale Part III (UPDRS III). The UPDRS III includes 31 items to evaluate motor disability, including speech, tremor at rest, rigidity, and posture [31]. The possible UPDRS III scores ranged from 0 to 108. A higher score indicated more severe motor disability. Disease duration was calculated as the number of months from the time PD was diagnosed to the present.

#### Nonmotor Symptoms of PD

A total of 5 nonmotor symptoms commonly reported in people with PD were measured: pain, constipation, dysphagia, sleep disturbances, and fatigue [24]. The severity of pain was measured using a combination of the Faces Pain Rating Scale and the visual analog scale. The possible scores ranged from 0 to 10, and a higher number indicated more severe pain. The participants could record their pain by dragging a slider on the screen with their fingers and by leaving notes about the pain sites. To measure the severity of constipation, participants were asked to record the frequency of defecation and click on a picture corresponding to the stool type. For better understanding, we provided example pictures representing 7 types of stools, based on the Bristol Stool Form Scale; the type-1 picture indicated severe constipation and the type-7 picture indicated severe diarrhea [32].

The degree of dysphagia was measured by clicking on a picture corresponding to the viscosity of food consumed. Example pictures showing 3 levels of food viscosity were provided based on the Fork Test. Level 1 indicated that the food did not flow off a fork and level 3 indicated that the food could not remain on a fork [33]. Sleep disturbances were measured using the Parkinson Disease Sleep Scale, which assesses sleep problems (eg, restlessness and hallucinations) frequently reported in people with PD [34]. The scale consisted of 15 items with total scores ranging from 0 to 150. A higher score indicated more severe sleep disturbances. The Parkinson Disease Fatigue Scale was used to measure the degree of fatigue [35]. Each item was scored from 1 to 5, and the sum of scores on 16 items indicated the degree of fatigue. A higher score meant more severe fatigue.

#### Self-Care

The degree of self-care was measured using the Self-Care of Chronic Illness Inventory (SC-CII) [36]. We used the Korean version of the SC-CII translated by Dr. Lee with permission from the original author. The Korean version of the SC-CII is downloadable from a website [37]. The SC-CII measures three domains of self-care: self-care maintenance (8 items), self-care monitoring (5 items), and self-care management (7 items). Each item was scored using a 5-point Likert scale (1=never or not likely, 5=always or very likely) with total scores ranging from 0 to 100. In previous studies, a total score of 70 or higher on the SC-CII was reported to indicate a high level of self-care [36,38]. The high validity and reliability of the SC-CII have been reported in previous research [38]. In this study, Cronbach α of the SC-CII was 0.580-0.673 in maintenance, 0.872-0.916 in monitoring, and 0.601-0.725 in management.

#### Satisfaction with the Yon PD App

To measure satisfaction with the Yon PD app, we developed a self-report questionnaire. The questionnaire consisted of 18 items asking about the user’s experience with the Yon PD app. Example questions included “Was the application easy to use?” “Was it easy to find the information you needed in the application?” “Has the application helped you improve your health?” and “Has the application improved access to healthcare service?” Each item was scored using the 7-point Likert scale (1=not likely, 7=very likely). The score totals ranged from 18 to 126, and a higher score indicated a greater satisfaction.

### Data Analysis

Data analysis was performed using SPSS version 26.0 (IBM Corp). Before the data analysis, missing values and extreme outliers were screened. The listwise deletion method was applied to handle missing values, and extreme outliers were deleted. The normal distribution of continuous data was checked using the Shapiro-Wilk test.

The general characteristics of the participants were analyzed using descriptive statistics, including mean, SD, frequency, and proportion. The homogeneity of 2 groups at baseline (general characteristics and self-care) was analyzed using the chi-square test, Fisher exact test, and independent, 2-tailed *t* test with an α level of .05. The effect of the intervention on self-care was analyzed using the repeated-measures ANOVA with an α level of .05. Satisfaction with the intervention was analyzed using mean and SD.

### Ethical Considerations

Ethical approval was obtained from the Institute Review Board of Yonsei University Health System Human Research Protection Center (Y-2020-0220). The hospital where this study was conducted belongs to the institution that granted ethical approval. Researchers explained the purpose and process of the study to eligible participants and obtained informed consent from each participant. Participants were guaranteed the right to withdraw, anonymity, and confidentiality of the collected data. All participants were compensated US $10 for each data collection. Participants who participated in all three data collection sessions were eligible to receive a total of US $30.

## Results

### General Characteristics

A total of 110 people with PD were enrolled in this study. Eight were excluded because they did not meet the inclusion criteria. Of the remaining people with PD, 48 were assigned to the intervention group and 54 to the control group. A total of 7 individuals in the intervention group and 2 in the control group were missing during the follow-up period. The missing rate was higher in the intervention group than in the control group. When 2 of the intervention group participants wanted to withdraw from the study, we asked them about the reason for their request, and they stated that they found the researchers’ contact bothersome.

**Figure 1:**
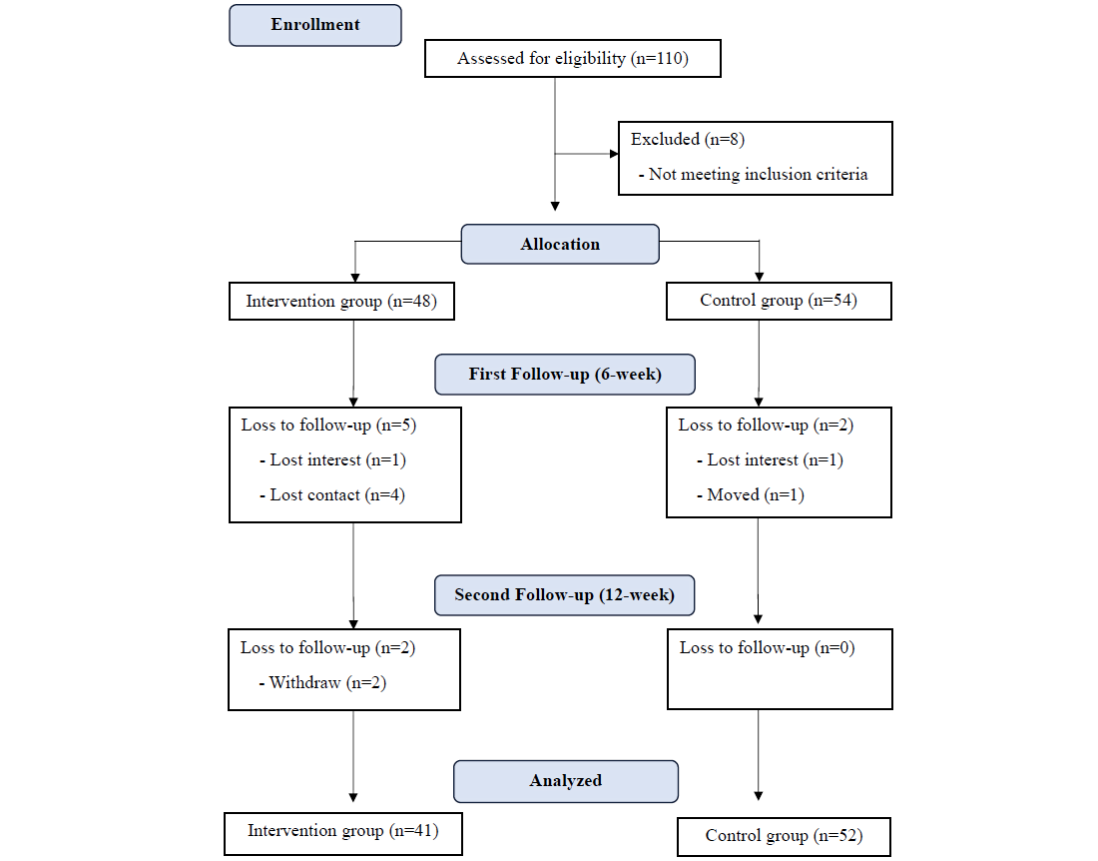
CONSORT flow diagram of enrollment.

A total of 93 people with PD were included in the analysis (41 in the intervention group and 52 in the control group; Figure 1). The homogeneity results for general characteristics are presented in Table 1. Between the 2 groups, there were no statistically significant differences in sex, age, income, marital status, education level, caregiver presence, living arrangement, employment, disease severity, disease duration, and number of comorbidities (all *P*>.05). In addition, the degree of self-care at baseline was not statistically different between the 2 groups (all *P*>.05).

**Table 1:** Homogeneity of general characteristics in 2 groups with Parkinson disease (n=93).

Variables		Experimental group^a^ (n=41)	Control group (n=52)		*χ*^2^ or *t* test *(df)*			*P* value		
**Sex, n (%)**							1.7 (1)^b^			.29^c^
	Male	14 (34.1)		24 (46.2)						
	Female	27 (65.9)	28 (53.8)							
Age (years), mean (SD)		62.20 (8.23)	62.19 (6.25)		–0.002 (91)^d^			>.99		
**Income per month (US $), n (%)**							0.1 (2)^b^			>.99^c^
	<2000	25 (61)	31 (59.6)							
	2000-2990	5 (12.2)	7 (13.5)							
	>3000	11 (26.8)	14 (26.9)							
**Marital status, n (%)**							4.3 (5)^b^			.35^c^
	Single	2 (4.9)	0 (0)							
	Married	32 (78.0)	45 (86.5)							
	Cohabitation	0 (0)	0 (0)							
	Widow or widower	2 (4.9)	3 (5.8)							
	Separation	0 (0)	1 (1.9)							
	Divorced	5 (12.2)	3 (5.8)							
**Education, n (%)**							2.8 (6)^b^			.82^c^
	No education	0 (0)	1 (1.9)							
	Elementary school	1 (2.4)	1 (1.9)							
	Middle school	6 (14.6)	6 (11.5)							
	High school	17 (41.5)	28 (53.8)							
	University or college	13 (31.7)	12 (23.1)							
	Graduate school	4 (9.8)	4 (7.7)							
**Caregiver, n (%)**							1.6 (1)^b^			.32^c^
	No	3 (7.3)	1 (1.9)							
	Yes	38 (92.7)	51 (98.1)							
**Living arrangements, n (%)**							3.4 (2)^b^			.20^c^
	Reside apart from the caregiver	0 (0)	3 (5.8)							
	Reside with the caregiver	38 (92.7)	48 (92.3)							
	No caregiver	3 (7.3)	1 (1.9)							
**Employment, n (%)**							2.9 (2)^b^			.24^c^
	Unemployed	18 (43.9)	24 (46.2)							
	Employed	15 (36.6)	24 (46.2)							
	Retired	8 (19.5)	4 (7.7)							
**Disease severity (UPDRS III^e^)**							0.804 (87)^d^			.42
	Provided records, n (%)	40 (97.6)	49 (94.2)							
	No records, n (%)	1 (2.4)	3 (5.8)							
	Score, mean (SD)	22.37 (10.61)	20.55 (10.49)							
Disease duration (months), mean (SD)		85.21 (56.61)	75.78 (63.68)		0.755 (91)^d^			.45		
**Number of comorbidities, n (%)**							3.8 (7)^b^			.76^c^
	None	16 (39)	22 (42.3)							
	1	16 (39)	15 (28.8)							
	2	6 (14.6)	5 (9.6)							
	3	1 (2.4)	4 (7.7)							
	4	1 (2.4)	3 (5.8)							
	5	1 (2.4)	2 (3.8)							
	6	0 (0)	0 (0)							
**Self-care (SC-CII^f^), mean (SD)**										
	Maintenance	58.78 (12.27)	60.67 (11.09)		0.779 (91)^d^			.44		
	Monitoring	60.57 (14)	60.77 (13.36)		0.070 (91)^d^			.94		
	Management	59.92 (12.19)	60.06 (11.42)		0.059 (91)^d^			.95		

^a^Testing a mobile app to monitor self-care.

^b^Chi-square test.

^c^Fisher exact test.

^d^*t* test.

^e^UPDRS III: Unified Parkinson’s Disease Rating Scale part III.

^f^SC-CII: self-care of chronic illness inventory.

### Effect of the Yon PD App on Self-Care

**Figure 2:**
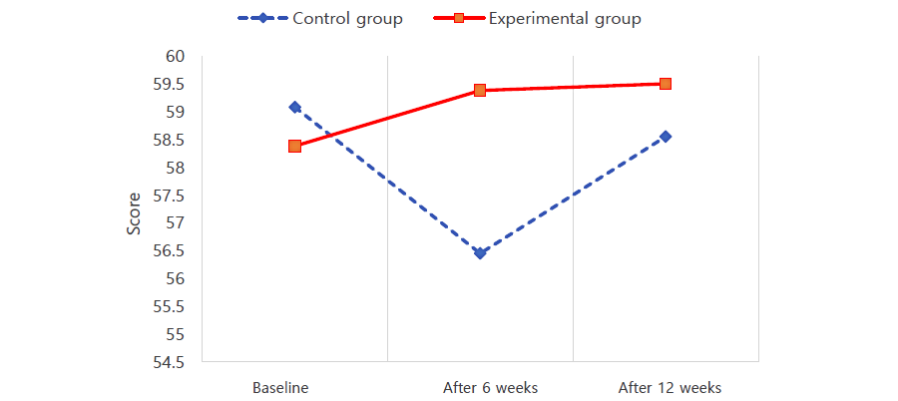
Effect of using a mobile app for self-care maintenance in 2 groups with Parkinson disease.

**Figure 3:**
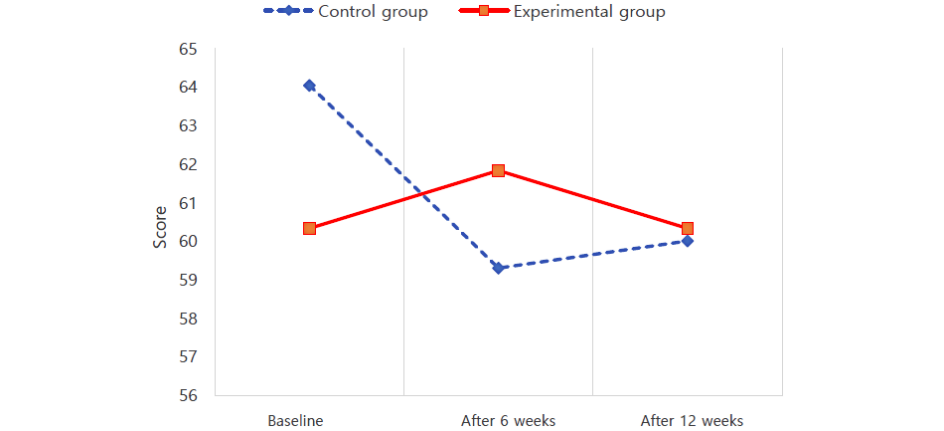
Effect of using a mobile app for self-care monitoring in 2 groups with Parkinson disease.

**Figure 4:**
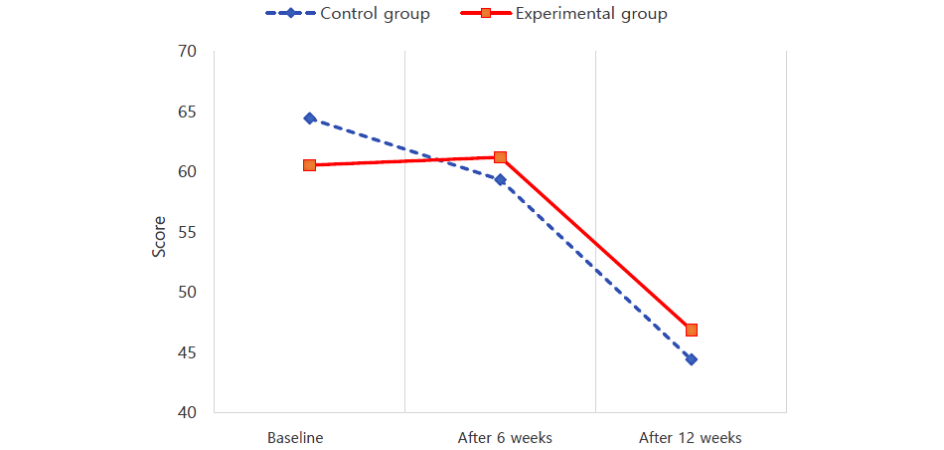
Effect of using a mobile app for self-care management in 2 groups with Parkinson disease.

The results of the repeated-measures ANOVA are presented in Table 2. Scores for self-care maintenance satisfied the Mauchly test of sphericity, a test that confirms the assumptions of repeated-measures ANOVA (W=0.989; *χ*^2^_2_=1.0; *P*=.61). Regarding self-care maintenance, there was a statistically significant interaction effect between groups and time (*F*_2182_=4.087; *P*=.02). Thus, using the Yon PD app was effective in increasing self-care maintenance (Figure 2). Scores for self-care monitoring also satisfied the Mauchly test (W=0.953; *χ*^2^_2_=4.3; *P*=.11), and there was a statistically significant interaction effect between groups and time on self-care monitoring (*F*_2182_=3.155; *P*=.045). Scores for self-care monitoring decreased slightly at 12 weeks compared to the baseline, but using the Yon PD app was effective in maintaining the level of self-care monitoring (Figure 3). Scores for self-care management also satisfied the Mauchly test of sphericity (W=0.980; *χ*^2^_2_=1.8; *P*=.40). In the 2 groups, self-care management scores changed depending on the measurement period. However, there was no statistically significant interaction effect between groups and time (*F*_2182_=1.348; *P*=.26). Therefore, using the Yon PD app was ineffective in increasing self-care management (Figure 4).

**Table 2:** Effect of the Yon PD app on self-care in people with Parkinson disease^a^ (n=93; experimental group, with app: n=41; control group, without app: n=52).

Variables groups		Baseline, mean (SD)	6 weeks, mean (SD)	12 weeks, mean (SD)	Sources	*F* or *t* test *(df)*	*P* value
**Maintenance**							
	Experimental	58.78 (12.27)	61.71 (10.86)	60.18 (10.34)	Group	.776 (191)^b^	.38
	Control	60.67 (11.09)	57.55 (10.22)	57.31 (12.08)	Time	.469 (2182)^c^	.63
	Group×time	—^d^	—	—	—	4.087 (2182)^c^	.02
**Monitoring**							
	Experimental	60.57 (14)	64.15 (15.29)	59.35 (13.85)	Group	1.277 (191)^b^	.26
	Control	60.77 (13.36)	57.50 (14.46)	56.92 (16.58)	Time	2.408 (2182)^c^	.09
	Group×time	—	—	—	—	3.155 (2182)^c^	.045
**Management**							
	Experimental	59.92 (12.19)	61.71 (13.95)	44.96 (11.79)	Group	.795 (191)^b^	.38
	Control	60.06 (11.42)	57.56 (12.35)	43.33 (12.78)	Time	95.051 (2182)^c^	<.001
	Group×time	—	—	—	—	1.348 (2182)^c^	.26

^a^Analyzed using the repeated-measures ANOVA.

^b^*t* test.

^c^*F* test.

^d^Not applicable.

### Adherence Rate and Satisfaction with the Yon PD App

The adherence rate was calculated as the total number of records divided by the number of required records. The overall 12-week adherence rate was 51.36% in the intervention group (n=41) and 56.2% in the control group (n=52). In the intervention group, the adherence rate was 60.84% at 6 weeks from baseline; the adherence rate gradually decreased after 6 weeks and was reported to be 41.87% at 12 weeks. A similar pattern was found in the control group; the adherence rate was 63.24% at 6 weeks and 51.37% at 12 weeks.

Among the 41 people with PD in the intervention group, 22 reported satisfaction with the Yon PD app. The average satisfaction score was 81.14 SD (24.46). The possible total scores ranged from 18 to 126; therefore, the average score was in the moderate to high range. Relatively low average scores were reported for items 9 and 16: “Did you feel comfortable using the application anywhere?” and “Does the application have all the features and requirements you expected?” In contrast, relatively high average scores were given to items 3 and 5: “Was it convenient to operate the screen?” and “If you made a mistake while using the application, were you able to correct it easily and quickly?” Detailed information on each item and the average scores are presented in Multimedia Appendix 1.

When investigating satisfaction with the Yon PD app, we also received subjective feedback from participants. Participants provided positive feedback, highlighting that the app’s interface was intuitive and enabled systematic symptom management (Table 3). Participants also provided feedback on what should be improved for the better use of the app. Most feedback was about technical issues while using the app, such as fast screen transitions, inconvenience with the auto-logout feature, the sensitivity of graphs when dragging the screen, and unstable internet connections. Some participants commented that it was tedious to record their symptoms because their symptoms did not substantially change during the participation; they also commented that they were bored with the app because they already knew the information it provided (Table 3).

**Table 3:** Subjective feedback from participants.

Category	Quotes
Positive experience	“The layout was easy to understand. The icons were clearly displayed with pictures, making it easy to determine what to click.” (Participant 5) “Using the app was convenient to record symptoms. I always carry my phone with me, so I could immediately record my symptoms whenever I experienced them.” (Participant 11)
Suggestions for improvement	“For me, using the app was boring because I already knew all the information provided in this app.” (Participant 6) “The app was automatically logged out if I did not use it for 30 minutes. I have to log-in again every time I want to do or record something.” (Participant 9) “My biggest problem was the internet connection. I don’t know why but the app was unstable on my phone and the internet connection was also unstable.” (Participant 17) “Recording symptoms twice a week was meaningless for me because the changes in symptoms were too minimal.” (Participant 20)

## Discussion

### Principal Findings

This study investigated whether monitoring the nonmotor symptoms of PD with the Yon PD app affected self-care over a 12-week period. As mentioned in the introduction, we had previously developed the Yon PD app and completed a 6-week feasibility study [24,25]. A notable difference in the current study was that the effectiveness of the Yon PD app was tested on a larger number of people with PD over a longer period. This study also contributed to the provision of advanced mobile health care services to people with PD by assessing adherence rates and satisfaction with the Yon PD app.

The people with PD involved in this study had a moderate level of self-care maintenance, monitoring, and management at baseline. Our findings demonstrated that monitoring the nonmotor symptoms of PD using the Yon PD app could be helpful in promoting self-care maintenance and monitoring. This means that people with PD pursued health behaviors to maintain their health and observed changes in symptoms while using the app. In the early stages of chronic diseases, it is important to monitor symptoms to identify problematic issues and rapidly respond to them. Regular symptom monitoring during the early stage of the disease can contribute to delaying disease progression and worsening conditions [39]. Self-care maintenance is also essential in chronic disease management. Pursuing health behaviors reduces the risk of comorbidities and strengthens the treatment effect [40]. In this study, it is speculated that the visualized history of nonmotor symptoms recorded in the app helped people with PD focus on their health. Recording symptoms on paper offers less visual stimulation, while the app’s visual features seem to have captured the attention of people with PD regarding their health.

Notably, self-care management gradually decreased in the intervention group despite their access to the Yon PD app; however, this trend was also found in the control group. A possible explanation is that 12 weeks constituted an insufficient time to change individuals’ behaviors. Given the older age of participants, they may need more time to change behaviors that have been ingrained for a long time [41]. Another possible explanation for these results may be individuals’ lack of motivation to change their health behaviors. The information-motivation-behavioral skills model posits that an individual’s behaviors are affected by information, motivation, and behavioral skills [42]. If the app could have stimulated the motivation of people with PD with new educational content or more structured feedback, behavior changes might have occurred.

In previous studies, motivational interviewing has been widely used to promote self-care in populations with chronic diseases. Motivational interviews are feasible using various methods, but face-to-face meetings were shown to be more effective than other methods in some studies [43]. In a randomized controlled trial that examined the effect of the motivational interview, patients with heart failure were given the opportunity to build supportive relationships with health care professionals and discuss their challenges [44]. For patients who had difficulty changing behaviors, the authors praised them whenever they made small behavioral changes. When the motivational interview was implemented over 1 year, these strategies effectively improved patients’ self-care in all domains [44]. To maximize the self-care effect of the Yon PD app, individuals should be motivated to use the app. Regularly scheduled follow-up calls, motivational interviews, and adding fun games to the app could be options to increase motivation [41,45].

In the intervention group, self-care monitoring scores increased for 6 weeks but decreased thereafter. It is speculated that the changes in self-care monitoring might be related to the rate of compliance with the Yon PD app. Like the self-care monitoring scores, adherence rates increased over 6 weeks and then decreased thereafter. Because the intervention group monitored nonmotor symptoms using the Yon PD app, poor adherence to the Yon PD app may lead to decreased interest in self-care monitoring. According to a study that reviewed adherence to technology-based interventions in older adults, a lack of motivation and enjoyment could be one of the reasons for a decreased adherence rate [46]. Thus, stimulating motivation to use the intervention and providing the intervention in an enjoyable format are important for increasing adherence rates. Providing tailored advice and ambient information regarding the patient’s particular nonmotor symptoms, as well as encouraging a positive experience with the app that helps them engage more actively, could be good strategies [47]. Overall, satisfaction with the Yon PD app was moderate to high. The average score for satisfaction was relatively low in 2 items asking, “Did you feel comfortable using the application anywhere?” and “Does the application have all the features and requirements you expected?” Given subjective feedback from participants, technical issues including unstable internet connections might have led to inconvenience. For participants who were knowledgeable about the disease, it could be frustrating to use the app because it provided educational information they already knew. To improve the user experience of the Yon PD app, ongoing software upgrades and content development are necessary.

### Limitations

This study has 2 main limitations. First, there can be sample bias because we limited the study participants to those who had and could use a smartphone. We chose a mobile app as the intervention delivery method in this study because smartphones are widely used worldwide, even among older adults. However, some people with PD may not be familiar with or own smartphones. This study did not include these populations, which limits the generalizability of the findings. Second, we only examined the perceived level of self-care. If actual behavioral changes had been objectively evaluated, the effectiveness of the Yon PD app could have been more extensively evaluated.

### Comparison With Previous Work

In previous studies, most mobile apps for people with PD have been designed to manage medication adherence and assess motor symptoms [21,22]. The management of these 2 types of symptoms is equally important; however, nonmotor symptoms have received less attention [23]. To fill this gap, we developed the Yon PD app, which allows users to record and monitor nonmotor symptoms of PD. This intuitively designed app provides exemplary pictures to help users understand. In addition, users can easily record their nonmotor symptoms by dragging score bars or clicking the image. The findings of this study indicate that using the Yon PD app to manage nonmotor symptoms can help people with PD improve self-care.

The Yon PD app can be applied in clinical and community settings. People with PD can use the app anywhere with an internet connection. Health care professionals can monitor nonmotor symptoms of people with PD through records sent from the app and provide feedback on self-care based on the data. These benefits allow continuous health care management regardless of time and distance.

### Conclusions

A high level of self-care is important for health maintenance in populations with chronic diseases. To promote self-care in people with PD, we examined the effectiveness of the Yon PD app, a mobile app that can monitor and record the nonmotor symptoms of PD. The Yon PD app was effective in increasing self-care maintenance and preventing a decline in self-care monitoring. However, it was not effective in improving self-care management. The failure of the app to improve self-care management seemed to be related to the relatively low app adherence rate. To increase the app adherence rate, additional strategies are required, including motivating users to use the app, providing new educational content, and organizing entertaining content. Future research should investigate the effectiveness of the revised app content in improving self-care management and the app adherence rate.

## Appendix

Multimedia Appendix 1 Satisfaction with the Yon PD app.

Multimedia Appendix 2 CONSORT-eHEALTH checklist (V 1.6.2).

## Abbreviations

CONSORT: Consolidated Standards of Reporting Trials
PD: Parkinson disease
SC-CII: Self-Care of Chronic Illness Inventory
UPDRS III: The Unified Parkinson’s disease Rating Scale Part III

## Data Availability

The datasets generated or analyzed during this study are not publicly available due to patients’ privacy but are available from the corresponding author on reasonable request.
