# Supplementary material for: Effect of the Yon PD App on the Management of Self-Care in People With Parkinson Disease: Randomized Controlled Trial
Source: J Med Internet Res. 2025 Apr 2;27:e62822. doi: 10.2196/62822 (PMC12004019; doi:10.2196/62822)
Supplement: Multimedia Appendix 1 [file jmir_v27i1e62822_app1.docx]

Appendix A. Satisfaction with the Yon PD app

| No. | Item | Min | Max | M±SD |
| --- | --- | --- | --- | --- |
| 1. | Was the application easy to use? | 1 | 7 | 4.73±1.80 |
| 2. | Was it easy to learn how to use the application? | 2 | 7 | 5.27±1.42 |
| 3. | Was it convenient to operate the screen? | 3 | 7 | 5.00±1.15 |
| 4. | Were you able to use all the functions provided by the application through the screen? | 1 | 7 | 4.86±1.46 |
| 5. | If you made a mistake while using the application, were you able to correct it easily and quickly? | 1 | 7 | 5.05±1.59 |
| 6. | Were you satisfied with the application screen layout? | 2 | 7 | 4.59±1.50 |
| 7. | Was it easy to find the information you needed in the application? | 1 | 7 | 4.32±1.62 |
| 8. | Did the application provide appropriate feedback on your activity progress? | 1 | 7 | 4.45±1.63 |
| 9. | Did you feel comfortable using the application anywhere? | 1 | 7 | 3.95±1.76 |
| 10. | Was the time spent using the application appropriate? | 2 | 7 | 4.95±1.33 |
| 11. | Would you like to use the application again? | 1 | 7 | 4.14±1.91 |
| 12. | Are you satisfied with the application overall? | 1 | 7 | 4.23±1.88 |
| 13. | Has the application helped your well-being? | 2 | 7 | 4.27±1.75 |
| 14. | Has the application improved access to healthcare service? | 1 | 7 | 4.36±1.76 |
| 15. | Has the application helped you improve your health? | 1 | 7 | 4.50±1.74 |
| 16. | Does the application have all the features and requirements you expected? | 1 | 7 | 3.77±1.69 |
| 17. | Was it possible to use the application without an internet connection? | 1 | 7 | 4.50±1.65 |
| 18. | Did the application provide an appropriate way to receive medical services? | 1 | 7 | 4.18±1.59 |
|  | Total |  |  | 81.14±24.46 |

M=mean; SD=standard deviation
