# Supplementary material for: Effect of the Yon PD App on the Management of Self-Care in People With Parkinson Disease: Randomized Controlled Trial
Source: J Med Internet Res. 2025 Apr 2;27:e62822. doi: 10.2196/62822 (PMC12004019; doi:10.2196/62822)
Supplement: Multimedia Appendix 2 [file jmir_v27i1e62822_app2.pdf]

CONSORT-EHEALTH Checklist V1.6.2 Report

Manuscript Number 62822

(based on CONSORT-EHEALTH V1.6), available at [http://tinyurl.com/consort-ehealth-v1-6].

Date completed

3/30/2025 22:33:50

by

Jee-Hye Yoo

Effect of the Yon PD App on the Management of Self-Care in People With Parkinson Disease: Randomized Controlled Trial

TITLE

1a-i) Identify the mode of delivery in the title

Effect of the Yon PD App on the Management of Self-Care in People With Parkinson Disease: Randomized Controlled Trial

1a-ii) Non-web-based components or important co-interventions in title

1a-iii) Primary condition or target group in the title

People With Parkinson Disease

ABSTRACT

1b-i) Key features/functionalities/components of the intervention and comparator in the METHODS section of the ABSTRACT

In our previous study, we developed a mobile app (Yon PD app) to monitor nonmotor symptoms of PD. In this study, we investigated the long-term effects of the app in a larger group of people. This was a randomized controlled trial. People with PD aged ≥50 years and able to use a smartphone were recruited from the neurology outpatient clinic of a tertiary hospital in South Korea.

1b-ii) Level of human involvement in the METHODS section of the ABSTRACT

1b-iii) Open vs. closed, web-based (self-assessment) vs. face-to-face assessments in the METHODS section of the ABSTRACT

1b-iv) RESULTS section in abstract must contain use data

In total, 93 participants were included in the analysis. There were 41 and 52 participants in the intervention and control groups, respectively. The general characteristics of the 2 groups were comparable. Monitoring nonmotor symptoms with the app effectively increased self-care maintenance (F2182=4.087; P=.02) and prevented a decrease in self-care monitoring (F2182=3.155;P=.045). However, using the app was ineffective in improving self-care management (F2182=1.348; P=.26). Self-care management gradually decreased over the 12-week period in both groups. The intervention adherence rate reached 60.84% at 6 weeks but
decreased to 41.87% by 12 weeks.

1b-v) CONCLUSIONS/DISCUSSION in abstract for negative trials

Participants were able to improve the degree of self-care by monitoring their nonmotor symptoms using the app. However, additional strategies that increase motivation and enjoyment are required to improve adherence.

INTRODUCTION

2a-i) Problem and the type of system/solution

Please see the introduction part

2a-ii) Scientific background, rationale: What is known about the (type of) system

Please see the introduction part

Does your paper address CONSORT subitem 2b?

Please see the introduction part

METHODS

3a) CONSORT: Description of trial design (such as parallel, factorial) including allocation ratio

Please see the methods part (sutdy desin, participants and sample size)

3b) CONSORT: Important changes to methods after trial commencement (such as eligibility criteria), with reasons

There were no chagnes during the study process.

3b-i) Bug fixes, Downtimes, Content Changes

4a) CONSORT: Eligibility criteria for participants

Please see figure 1 CONSORT flow diagram

4a-i) Computer / Internet literacy

4a-ii) Open vs. closed, web-based vs. face-to-face assessments:

Participants were recruited through advertisements and referrals within the hospital from October 2022 to February 2023.

4a-iii) Information giving during recruitment

Please see the method section (study process)

4b) CONSORT: Settings and locations where the data were collected

Please see the methods (sutdy desin)

4b-i) Report if outcomes were (self-)assessed through online questionnaires

Please see the result section

4b-ii) Report how institutional affiliations are displayed

5) CONSORT: Describe the interventions for each group with sufficient details to allow replication, including how and when they were actually administered

5-i) Mention names, credential, affiliations of the developers, sponsors, and owners

5-ii) Describe the history/development process

Please see the method seciton

5-iii) Revisions and updating

Please see the result section

5-iv) Quality assurance methods

5-v) Ensure replicability by publishing the source code, and/or providing screenshots/screen-capture video, and/or providing flowcharts of the algorithms used

5-vi) Digital preservation

5-vii) Access

the information is described in the method section

5-viii) Mode of delivery, features/functionalities/components of the intervention and comparator, and the theoretical framework

Please see the method section (study process)

5-ix) Describe use parameters

Please see the method section (intervention group, study process)

5-x) Clarify the level of human involvement

**5-xi) Report any prompts/reminders used**		
Please see the method section (study process)		
**5-xii) Describe any co-interventions (incl. training/support)**		
There was no co-interventions in this study.		
**6a) CONSORT: Completely defined pre-specified primary and secondary outcome measures, including how and when they were assessed**		
Please see the method section (study process)		
**6a-i) Online questionnaires: describe if they were validated for online use and apply CHERRIES items to describe how the questionnaires were designed/deployed**		
**6a-ii) Describe whether and how “use” (including intensity of use/dosage) was defined/measured/monitored**		
Please see the method section		
**6a-iii) Describe whether, how, and when qualitative feedback from participants was obtained**		
**6b) CONSORT: Any changes to trial outcomes after the trial commenced, with reasons**		
Please see the methods (sutdy desin)		
**7a) CONSORT: How sample size was determined**		
**7a-i) Describe whether and how expected attrition was taken into account when calculating the sample size**		
The sample size was calculated using the G*Power 3.1 program(Heinrich-Heine-Universität)		
**7b) CONSORT: When applicable, explanation of any interim analyses and stopping guidelines**		
Please see the method section (study process)		
**8a) CONSORT: Method used to generate the random allocation sequence**		
The generated random numbers were matched sequentially to the enrolled participants. If the last digit of the number was even, the participant was assigned to the intervention group; otherwise, the participant was assigned to the control group.		
**8b) CONSORT: Type of randomisation; details of any restriction (such as blocking and block size)**		
The generated random numbers were matched sequentially to the enrolled participants. If the last digit of the number was even, the participant was assigned to the intervention group; otherwise, the participant was assigned to the control group.		
**9) CONSORT: Mechanism used to implement the random allocation sequence (such as sequentially numbered containers), describing any steps taken to conceal the sequence until interventions were assigned**		
The generated random numbers were matched sequentially to the enrolled participants. If the last digit of the number was even, the participant was assigned to the intervention group; otherwise, the participant was assigned to the control group.		
**10) CONSORT: Who generated the random allocation sequence, who enrolled participants, and who assigned participants to interventions**		
The participants were randomly assigned to either an intervention or control group using computer-generated random numbers.		
**11a) CONSORT: Blinding - If done, who was blinded after assignment to interventions (for example, participants, care providers, those assessing outcomes) and how**		
**11a-i) Specify who was blinded, and who wasn’t**		
During the study, researchers did not notify the participants whether they were in the intervention or control group. Thus, only the participants were blinded.		
**11a-ii) Discuss e.g., whether participants knew which intervention was the “intervention of interest” and which one was the “comparator”**		
**11b) CONSORT: If relevant, description of the similarity of interventions**		
Not applicable in this study		
**12a) CONSORT: Statistical methods used to compare groups for primary and secondary outcomes**		
Please see the data analysis part		
**12a-i) Imputation techniques to deal with attrition / missing values**		
The listwise deletion method was applied to handle missing values, and extreme outliers were deleted.		
**12b) CONSORT: Methods for additional analyses, such as subgroup analyses and adjusted analyses**		
Satisfaction with the intervention was analyzed using mean and SD.		
RESULTS		
**13a) CONSORT: For each group, the numbers of participants who were randomly assigned, received intended treatment, and were analysed for the primary outcome**		
Please see the result section		
**13b) CONSORT: For each group, losses and exclusions after randomisation, together with reasons**		
Please see figure 1 CONSORT flow diagram		
**13b-i) Attrition diagram**		
Please see figure 1 CONSORT flow diagram		
**14a) CONSORT: Dates defining the periods of recruitment and follow-up**		
Please see the methods part and result part		
**14a-i) Indicate if critical “secular events” fell into the study period**		
**14b) CONSORT: Why the trial ended or was stopped (early)**		
This study was designed as a 12-week intervention study to investigate the long-term effects of the Yon PD app.		
**15) CONSORT: A table showing baseline demographic and clinical characteristics for each group**		
Table 1 demonstrates demographic characteristics of the intervention and control groups.		
**15-i) Report demographics associated with digital divide issues**		
Not applicable in this study.		
**16a) CONSORT: For each group, number of participants (denominator) included in each analysis and whether the analysis was by original assigned groups**		
**16-i) Report multiple “denominators” and provide definitions**		
We clearly defined N throughout the manuscript.		
**16-ii) Primary analysis should be intent-to-treat**		
**17a) CONSORT: For each primary and secondary outcome, results for each group, and the estimated effect size and its precision (such as 95% confidence interval)**		
Please see the result section and Table 1 & 2.		
**17a-i) Presentation of process outcomes such as metrics of use and intensity of use**		
**17b) CONSORT: For binary outcomes, presentation of both absolute and relative effect sizes is recommended**		
Please see table 1 & 2 in the result section.		
**18) CONSORT: Results of any other analyses performed, including subgroup analyses and adjusted analyses, distinguishing pre-specified from exploratory**		
The adherence rate was calculated as the total number of records divided by the number of required records.		
**18-i) Subgroup analysis of comparing only users**		
**19) CONSORT: All important harms or unintended effects in each group**		
Not applicable in this study. Potential harms were described in the ethical consideration part.		
**19-i) Include privacy breaches, technical problems**		
**19-ii) Include qualitative feedback from participants or observations from staff/researchers**		

DISCUSSION		
**20) CONSORT: Trial limitations, addressing sources of potential bias, imprecision, multiplicity of analyses**		
**20-i) Typical limitations in ehealth trials**		
This study has 2 main limitations. First, there can be sample bias because we limited the study participants to those who had and could use a smartphone.		
**21) CONSORT: Generalisability (external validity, applicability) of the trial findings**		
**21-i) Generalizability to other populations**		
**21-ii) Discuss if there were elements in the RCT that would be different in a routine application setting**		
**22) CONSORT: Interpretation consistent with results, balancing benefits and harms, and considering other relevant evidence**		
**22-i) Restate study questions and summarize the answers suggested by the data, starting with primary outcomes and process outcomes (use)**		
Please see the result section		
**22-ii) Highlight unanswered new questions, suggest future research**		
Other information		
**23) CONSORT: Registration number and name of trial registry**		
Clinical Research Information Service KCT0006433;<br>https://cris.nih.go.kr/cris/search/detailSearch.do?seq=19877&search_page=L		
**24) CONSORT: Where the full trial protocol can be accessed, if available**		
Not applicable in this study.		
**25) CONSORT: Sources of funding and other support (such as supply of drugs), role of funders**		
We provided funding information in metadata.		
**X26-i) Comment on ethics committee approval**		
Ethical approval was obtained from the Institute Review Board of a large tertiary hospital Human Research Protection Center(Y-2020-0220).		
**x26-ii) Outline informed consent procedures**		
**X26-iii) Safety and security procedures**		
Researchers explained the purpose and process of the study to eligible participants and obtained informed consent from each participant. Participants were guaranteed the right to withdraw, anonymity, and confidentiality of the collected data.		
**X27-i) State the relation of the study team towards the system being evaluated**		
